# Supplementary figures and images for: Evaluating the impact of differentiated service delivery (DSD) on retention in care and HIV viral suppression in South Africa: A target trial emulation using routine healthcare data
Source: PLoS Med. 2025 Aug 26;22(8):e1004489. doi: 10.1371/journal.pmed.1004489 (PMC12410879; doi:10.1371/journal.pmed.1004489)

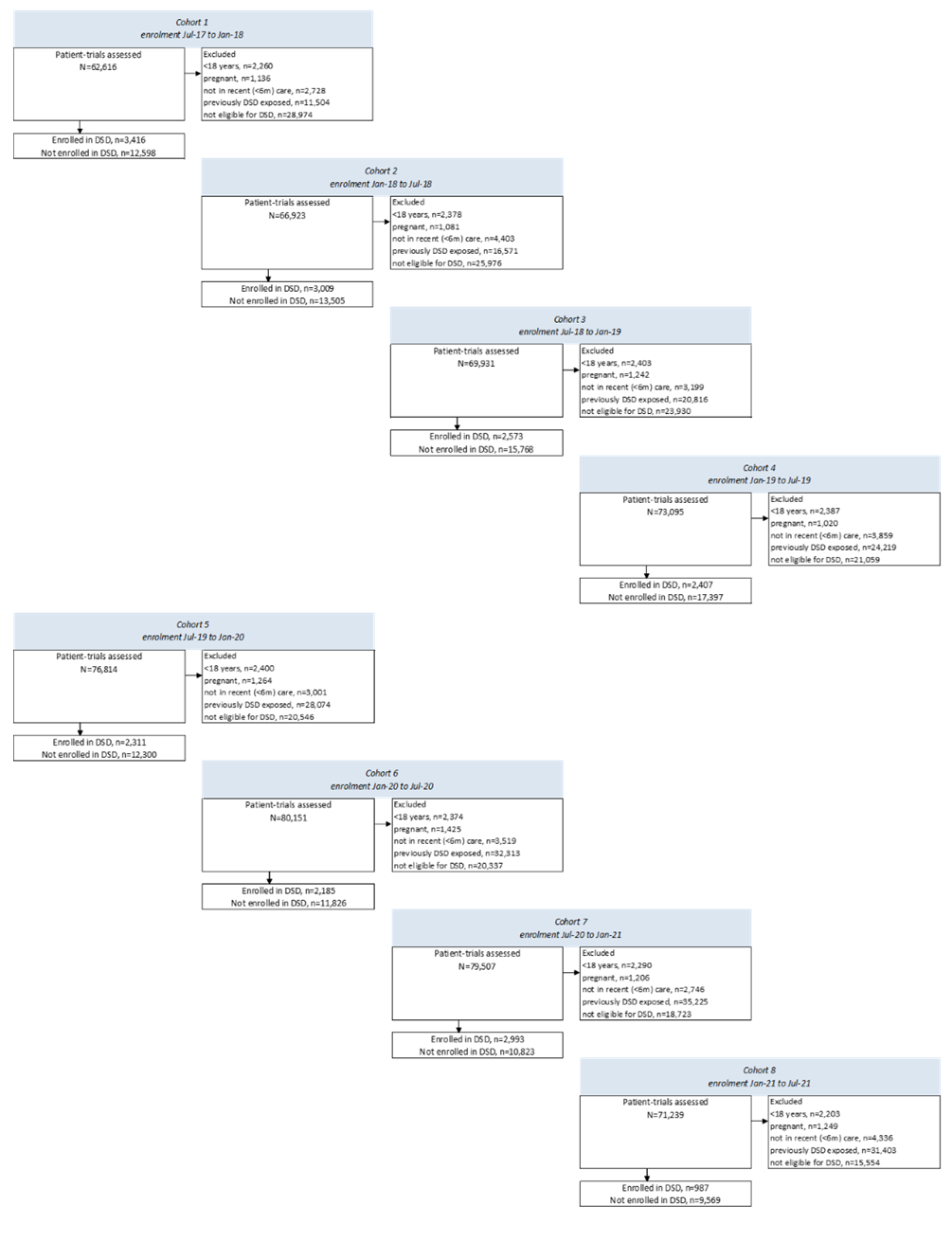

Supplement: S1 Fig — (TIF) [file pmed.1004489.s001.tif]
