# Supplementary material for: Evaluating the impact of differentiated service delivery (DSD) on retention in care and HIV viral suppression in South Africa: A target trial emulation using routine healthcare data
Source: PLoS Med. 2025 Aug 26;22(8):e1004489. doi: 10.1371/journal.pmed.1004489 (PMC12410879; doi:10.1371/journal.pmed.1004489)
Supplement: S1 Table — (DOCX) [file pmed.1004489.s002.docx]

**Table S1. Study site description**

| **Site** | **Setting** | **Number on ART 2021** | **Approximate % of ART clients enrolled in DSD models (2021)** |  |
| --- | --- | --- | --- | --- |
| *Ekurhuleni District (data from Jan 2016 - Jun 2020)* | | | | |
| Clinic | Urban | 2,386 | 53% |  |
| Clinic | Urban | 2,658 | 44% |  |
| Clinic | Urban | 7,213 | 51% |  |
| Clinic | Urban | 1,482 | Missing |  |
| Clinic | Urban | 3,502 | Missing |  |
| Clinic | Urban | 4,560 | Missing |  |
| *West Rand District (data from Jan 2016 - Jun 2023)* | | | | |
| Clinic | Urban | 1,783 | Missing |  |
| Clinic | Rural | 1,803 | 32% |  |
| Clinic | Urban | 1,897 | 24% |  |
| Clinic | Urban | 2,116 | 57% |  |
| Clinic | Rural | 2,301 | 43% |  |
| Clinic | Urban | 2,959 | 66% |  |
| *Ehlanzeni District (data from Jan 2016 - Jan 2023)* | | | | |
| Community Health Centre | Urban | 6,622 | 61% |  |
| Clinic | Rural | 3,553 | 44% |  |
| Clinic | Rural | 1,943 | 48% |  |
| Clinic | Rural | 3,001 | 11% |  |
| Community Health Centre | Urban | 5,234 | 25% |  |
| Clinic | Urban | 5,515 | 28% |  |
| *King Cetshwayo District (data from Jan 2016 - Apr 2023)* | | | | |
| Clinic | Rural | 1,182 | 24% |  |
| Clinic | Rural | 1,509 | 81% |  |
| Clinic | Rural | 2,231 | 78% |  |
| Clinic | Rural | 3,361 | Missing |  |
| Clinic | Rural | 5,190 | 61% |  |
| Clinic | Urban | 7,934 | 75% |  |
