# Supplementary material for: Evaluating the impact of differentiated service delivery (DSD) on retention in care and HIV viral suppression in South Africa: A target trial emulation using routine healthcare data
Source: PLoS Med. 2025 Aug 26;22(8):e1004489. doi: 10.1371/journal.pmed.1004489 (PMC12410879; doi:10.1371/journal.pmed.1004489)
Supplement: S2 Table — (DOCX) [file pmed.1004489.s003.docx]

**Table S2. Baseline demographics of trial-clients eligible for DSD models across all eight emulated target trials**

|  |  | **Total (%)** | **Not enrolled in DSD (%)** | **Enrolled in DSD (%)** |
| --- | --- | --- | --- | --- |
| **Number of trial-clients *** |  | **148,943** | **123,168** | **25,775** |
| Age group in years | 18-24 | 6,664 (4%) | 5,773 (87%) | 891 (13%) |
|  | 25-34 | 40,063 (27%) | 32,973 (82%) | 7,090 (18%) |
|  | 35-49 | 70,700 (47%) | 57,676 (82%) | 13,024 (18%) |
|  | 50+ | 31,516 (21%) | 26,746 (85%) | 4,770 (15%) |
| Sex | Female | 102,680 (69%) | 84,808 (83%) | 17,872 (17%) |
|  | Male | 46,263 (31%) | 38,360 (83%) | 7,903 (17%) |
| Years on ART at trial start,  median (IQR) |  | 4.1 (2.4 - 6.7) | 4.1 (2.4 - 6.7) | 4.0 (2.3 - 6.5) |
| Years on ART at trial start | 1-<2 | 26,590 (18%) | 21,674 (82%) | 4,916 (18%) |
|  | 2-<5 | 62,539 (42%) | 51,555 (82%) | 10,984 (18%) |
|  | 5+ | 59,814 (40%) | 49,939 (83%) | 9,875 (17%) |
| WHO stage at ART initiation | 1 | 86,361 (65%) | 70,812 (82%) | 15,549 (18%) |
|  | 2 | 25,732 (19%) | 21,428 (83%) | 4,304 (17%) |
|  | 3 | 17,901 (13%) | 14,895 (83%) | 3,006 (17%) |
|  | 4 | 2,842 (2%) | 2,390 (84%) | 452 (16%) |
| CD4 (cells/µl) at ART initiation; median (IQR) | | 238 (128 - 371) | 237 (128 - 372) | 239 (129 - 370) |
| Location | Rural | 42,292 (28%) | 35,896 (85%) | 6,396 (15%) |
|  | Urban | 106,651 (72%) | 87,272 (82%) | 19,379 (18%) |
| Province | Gauteng | 71,265 (48%) | 58,577 (82%) | 12,688 (18%) |
|  | KwaZulu-Natal | 37,327 (25%) | 31,830 (85%) | 5,497 (15%) |
|  | Mpumalanga | 40,351 (27%) | 32,761 (81%) | 7,590 (19%) |

* trial-clients refers to the number of clients followed across all 8 target trials, with some clients appearing in multiple target trials
