# Supplementary material for: Evaluating the impact of differentiated service delivery (DSD) on retention in care and HIV viral suppression in South Africa: A target trial emulation using routine healthcare data
Source: PLoS Med. 2025 Aug 26;22(8):e1004489. doi: 10.1371/journal.pmed.1004489 (PMC12410879; doi:10.1371/journal.pmed.1004489)
Supplement: S4 Table — (DOCX) [file pmed.1004489.s005.docx]

**Table S4. Pooled adjusted risk difference (%) of retention in care and viral suppression by DSD enrolment at 12, 24 and 36 months, adjusting for CD4 count at ART initiation**

|  |  | **Retention in care** | | | **Viral suppression** | | | |  |
| --- | --- | --- | --- | --- | --- | --- | --- | --- | --- |
|  |  | **Retained/N (%)** | **Unadjusted risk difference (95% confidence interval)** | **Adjusted* risk difference (95% confidence interval)** | | **Virally suppressed/N** (%)** | **Unadjusted risk difference (95% confidence interval)** | **Adjusted risk difference (95% confidence interval)** | |
| **12 months** |  |  |  |  | |  |  |  | |
| DSD enrolment | No | 93,966/103,786 (91%) | reference | reference | | 74,108/77,643 (95%) | reference | reference | |
|  | Yes | 18,583/19,881 (93%) | 2.9 (1.5,4.4) | 3.0 (1.3,4.7) | | 14,824/15,320 (97%) | 1.3 (-0.4,3.0) | 1.4 (-0.5,3.3) | |
| Age group (years) | 18-24 | 5,010/5,883 (85%) | -7.3 (-9.7,-4.7) | -6.2 (-9.2,-3.1) | | 3,788/4,096 (92%) | -3.6 (-6.6,-0.4) | -3.7 (-7.4,0.0) | |
|  | 25-34 | 30,506/34,203 (89%) | -3.2 (-4.5,-1.9) | -2.8 (-4.3,-1.3) | | 23,878/25,100 (95%) | -0.9 (-2.4,0.6) | -1.2 (-2.9,0.6) | |
|  | 35-49 | 53,175/57,542 (92%) | reference | reference | | 42,307/44,051 (96%) | reference | reference | |
|  | 50+ | 23,858/26,039 (92%) | -0.8 (-2.2,0.6) | -0.9 (-2.5,0.7) | | 18,959/19,716 (96%) | 0.1 (-1.5,1.8) | 0.2 (-1.6,2.1) | |
| Sex | Female | 78,225/85,960 (91%) | reference | reference | | 62,330/64,863 (96%) | reference | reference | |
|  | Male | 34,324/37,707 (91%) | 0.0 (-1.1,1.2) | -0.6 (-1.9,0.7) | | 26,602/28,100 (95%) | -1.4 (-2.8,-0.1) | -1.6 (-3.2,0.0) | |
| Location | Rural | 38,137/41,278 (92%) | reference | reference | | 29,723/31,548 (94%) | reference | reference | |
|  | Urban | 74,412/82,389 (90%) | -2.1 (-3.2,-0.9) | -1.9 (-3.6,-0.3) | | 59,209/61,415 (96%) | 2.2 (0.9,3.5) | 1.0 (-1.0,2.9) | |
| Province | GP | 42,750/47,423 (90%) | reference | reference | | 33,615/34,716 (97%) | reference | reference | |
|  | KZN | 33,793/37,065 (91%) | 1.0 (-0.3,2.3) | 1.1 (-0.5,2.7) | | 28,242/29,365 (96%) | -0.7 (-2.2,0.9) | 0.2 (-1.7,2.1) | |
|  | MP | 36,006/39,179 (92%) | 1.8 (0.5,3.0) | 0.7 (-1.1,2.6) | | 27,075/28,882 (94%) | -3.1 (-4.6,-1.6) | -2.6 (-4.8,-0.3) | |
| Years on ART | 1-<2 | 20,108/22,711 (89%) | -3.8 (-5.3,-2.3) | -2.7 (-4.5,-0.8) | | 16,016/16,806 (95%) | -0.6 (-2.4,1.2) | -0.5 (-2.7,1.7) | |
|  | 2-<5 | 47,995/52,818 (91%) | -1.5 (-2.6,-0.3) | -0.7 (-2.1,0.6) | | 37,571/39,298 (96%) | -0.3 (-1.7,1.1) | -0.2 (-1.8,1.5) | |
|  | 5+ | 44,446/48,138 (92%) | reference | reference | | 35,345/36,859 (96%) | reference | reference | |
| CD4 (cells/µl) at ART initiation | <200 | 36,949/40,107 (92%) | reference | reference | | 29,048/30,594 (95%) | reference | reference | |
|  | 200-349 | 27,694/30,341 (91%) | -0.9 (-2.3,0.6) | -0.5 (-2.0,0.9) | | 21,964/22,857 (96%) | 1.1 (-0.5,2.8) | 1.3 (-0.4,3.0) | |
|  | 350-499 | 13,619/15,004 (91%) | -1.4 (-3.1,0.4) | -0.5 (-2.3,1.4) | | 10,726/11,165 (96%) | 1.1 (-1.0,3.2) | 1.5 (-0.7,3.7) | |
|  | 500+ | 11,282/12,636 (89%) | -2.8 (-4.7,-0.9) | -1.6 (-3.6,0.4) | | 9,071/9,361 (97%) | 2.0 (-0.3,4.2) | 2.3 (0.0,4.7) | |
| **24 months** |  |  |  |  | |  |  |  | |
| DSD enrolment | No | 61,943/72,788 (85%) | reference | reference | | 48,637/51,107 (95%) | reference | reference | |
|  | Yes | 12,203/13,733 (89%) | 3.8 (2.1,5.5) | 3.8 (1.9,5.8) | | 9,943/10,241 (97%) | 1.9 (-0.2,4.0) | 1.8 (-0.5,4.1) | |
| Age group (years) | 18-24 | 3,102/4,031 (77%) | -10.7 (-13.6,-7.9) | -9.7 (-13.1,-6.1) | | 2,342/2,551 (92%) | -4.2 (-8.0,-0.3) | -3.8 (-8.5,0.9) | |
|  | 25-34 | 20,214/24,142 (84%) | -4.0 (-5.4,-2.5) | -3.5 (-5.2,-1.8) | | 15,752/16,652 (95%) | -1.4 (-3.3,0.4) | -1.8 (-3.9,0.3) | |
|  | 35-49 | 35,234/40,178 (88%) | reference | reference | | 28,080/29,248 (96%) | reference | reference | |
|  | 50+ | 15,596/18,170 (86%) | -1.9 (-3.5,-0.2) | -2.0 (-3.8,-0.1) | | 12,406/12,897 (96%) | 0.2 (-1.8,2.2) | 0.4 (-1.9,2.7) | |
| Sex | Female | 51,960/60,536 (86%) | reference | reference | | 41,421/43,186 (96%) | reference | reference | |
|  | Male | 22,186/25,985 (85%) | -0.5 (-1.8,0.9) | -1.3 (-2.8,0.3) | | 17,159/18,162 (94%) | -1.4 (-3.1,0.3) | -1.6 (-3.5,0.3) | |
| Location | Rural | 26,638/30,270 (88%) | reference | reference | | 20,544/21,884 (94%) | reference | reference | |
|  | Urban | 47,508/56,251 (84%) | -3.5 (-4.8,-2.2) | -3.1 (-5.0,-1.3) | | 38,036/39,464 (96%) | 2.5 (0.9,4.1) | 0.6 (-1.7,2.9) | |
| Province | GP | 24,846/29,616 (84%) | reference | reference | | 19,713/20,294 (97%) | reference | reference | |
|  | KZN | 23,537/27,290 (86%) | 2.4 (0.8,3.9) | 2.3 (0.4,4.1) | | 19,762/20,596 (96%) | -1.2 (-3.1,0.7) | -0.6 (-2.9,1.7) | |
|  | MP | 25,763/29,615 (87%) | 3.1 (1.6,4.6) | 1.7 (-0.4,3.8) | | 19,105/20,458 (93%) | -3.8 (-5.6,-1.9) | -3.6 (-6.2,-0.9) | |
| Years on ART | 1-<2 | 13,513/16,314 (83%) | -4.5 (-6.3,-2.8) | -3.2 (-5.3,-1.0) | | 10,648/11,197 (95%) | -0.9 (-3.1,1.3) | -0.9 (-3.6,1.8) | |
|  | 2-<5 | 32,288/37,761 (86%) | -1.9 (-3.2,-0.5) | -1.1 (-2.8,0.5) | | 25,282/26,561 (95%) | -0.8 (-2.5,0.9) | -1.0 (-3.0,1.0) | |
|  | 5+ | 28,345/32,446 (87%) | reference | reference | | 22,650/23,590 (96%) | reference | reference | |
| CD4 (cells/µl) at ART initiation | <200 | 24,666/28,327 (87%) | reference | reference | | 19,386/20,496 (95%) | reference | reference | |
|  | 200-349 | 18,965/22,058 (86%) | -1.1 (-2.7,0.5) | -0.7 (-2.4,0.9) | | 15,103/15,735 (96%) | 1.4 (-0.6,3.4) | 1.8 (-0.3,3.8) | |
|  | 350-499 | 9,030/10,559 (86%) | -1.6 (-3.6,0.5) | -0.5 (-2.6,1.7) | | 7,093/7,382 (96%) | 1.5 (-1.1,4.1) | 2.3 (-0.4,5.0) | |
|  | 500+ | 6,879/8,241 (83%) | -3.6 (-5.8,-1.3) | -2.1 (-4.4,0.3) | | 5,512/5,701 (97%) | 2.1 (-0.8,5.0) | 2.9 (-0.1,5.9) | |
| **36 months** |  |  |  |  | |  |  |  | |
| DSD enrolment | No | 33,568/41,512 (81%) | reference | reference | | 26,481/27,735 (95%) | reference | reference | |
|  | Yes | 6,810/8,015 (85%) | 4.1 (1.9,6.3) | 3.7 (1.3,6.2) | | 5,635/5,807 (97%) | 1.6 (-1.2,4.4) | 1.5 (-1.5,4.6) | |
| Age group (years) | 18-24 | 1,679/2,351 (71%) | -12.4 (-16.0,-8.7) | -11.6 (-15.8,-7.2) | | 1,273/1,372 (93%) | -3.3 (-8.6,2.1) | -2.8 (-9.0,3.7) | |
|  | 25-34 | 11,162/13,950 (80%) | -3.8 (-5.7,-1.9) | -3.4 (-5.6,-1.3) | | 8,792/9,259 (95%) | -1.2 (-3.6,1.4) | -1.4 (-4.3,1.4) | |
|  | 35-49 | 19,090/22,773 (84%) | reference | reference | | 15,356/15,978 (96%) | reference | reference | |
|  | 50+ | 8,447/10,453 (81%) | -3.0 (-5.1,-0.9) | -3.0 (-5.3,-0.6) | | 6,695/6,933 (97%) | 0.5 (-2.3,3.2) | 0.5 (-2.6,3.6) | |
| Sex | Female | 28,593/34,875 (82%) | reference | reference | | 23,010/23,910 (96%) | reference | reference | |
|  | Male | 11,785/14,652 (80%) | -1.6 (-3.3,0.2) | -2.5 (-4.4,-0.5) | | 9,106/9,632 (95%) | -1.7 (-4.0,0.6) | -1.9 (-4.5,0.7) | |
| Location | Rural | 16,004/18,965 (84%) | reference | reference | | 12,313/13,035 (94%) | reference | reference | |
|  | Urban | 24,374/30,562 (80%) | -4.6 (-6.3,-3.0) | -3.9 (-6.2,-1.7) | | 19,803/20,507 (97%) | 2.1 (0,4.2) | 0.8 (-2.1,3.7) | |
| Province | GP | 10,396/13,281 (78%) | reference | reference | | 8,349/8,578 (97%) | reference | reference | |
|  | KZN | 14,867/18,068 (82%) | 4.0 (2.0,6.0) | 3.5 (1.1,5.9) | | 12,485/12,969 (96%) | -1.1 (-3.8,1.6) | -0.5 (-3.7,2.7) | |
|  | MP | 15,115/18,178 (83%) | 4.9 (2.9,6.9) | 2.6 (-0.1,5.3) | | 11,282/11,995 (94%) | -3.3 (-6.0,-0.6) | -2.7 (-6.3,1.0) | |
| Years on ART | 1-<2 | 8,229/10,485 (78%) | -5.0 (-7.2,-2.9) | -3.7 (-6.3,-1.0) | | 6,524/6,854 (95%) | -1.2 (-4.0,1.7) | -1.2 (-4.7,2.3) | |
|  | 2-<5 | 17,295/21,256 (81%) | -2.1 (-4.0,-0.3) | -1.6 (-3.7,0.5) | | 13,644/14,286 (96%) | -0.8 (-3.2,1.5) | -1.0 (-3.7,1.7) | |
|  | 5+ | 14,854/17,786 (84%) | reference | reference | | 11,948/12,402 (96%) | reference | reference | |
| CD4 (cells/µl) at ART initiation | <200 | 13,478/16,254 (83%) | reference | reference | | 10,662/11,214 (95%) | reference | reference | |
|  | 200-349 | 10,881/13,225 (82%) | -0.6 (-2.7,1.4) | -0.3 (-2.4,1.8) | | 8,762/9,099 (96%) | 1.2 (-1.5,3.9) | 1.5 (-1.3,4.2) | |
|  | 350-499 | 4,991/6,161 (81%) | -1.9 (-4.5,0.7) | -0.8 (-3.5,2.0) | | 3,996/4,161 (96%) | 1.0 (-2.5,4.5) | 1.5 (-2.1,5.2) | |
|  | 500+ | 3,472/4,422 (79%) | -4.4 (-7.3,-1.4) | -2.7 (-5.8,0.4) | | 2,814/2,919 (96%) | 1.3 (-2.6,5.4) | 1.8 (-2.4,6.0) | |

*estimates adjusted for age, sex, urban/rural facility setting, province, WHO stage at ART initiation, years on ART at trial enrolment

**denominator only includes those with a viral load measured during the 12-, 24- or 36-month outcome periods
