# Supplementary material for: Evaluating the impact of differentiated service delivery (DSD) on retention in care and HIV viral suppression in South Africa: A target trial emulation using routine healthcare data
Source: PLoS Med. 2025 Aug 26;22(8):e1004489. doi: 10.1371/journal.pmed.1004489 (PMC12410879; doi:10.1371/journal.pmed.1004489)
Supplement: S5 Table — (DOCX) [file pmed.1004489.s006.docx]

**Table S5. Age-stratified pooled risk differences for retention**

| **Age group** | **n/N (%) retained**  **in DSD** | **n/N (%) retained**  **in non-DSD** | **Unadjusted Risk Difference comparing DSD vs non-DSD (95% CI)** | **Adjusted* Risk Difference comparing DSD vs non-DSD (95% CI)** |
| --- | --- | --- | --- | --- |
| **12 months** | | | | |
| 18-24 | 660/745 (89%) | 4,350/5,138 (85%) | 3.9 (-3.1,11.3) | 4.4 (-3.2,12.3) |
| 25-34 | 5,268/5,713 (92%) | 25,238/28,491 (89%) | 3.6 (0.9,6.4) | 4.1 (1.3,7.0) |
| 35-49 | 9,241/9,797 (94%) | 43,934/47,745 (92%) | 2.3 (0.2,4.4) | 2.5 (0.3,4.8) |
| 50+ | 3,414/3,627 (94%) | 20,444/22,412 (91%) | 2.9 (-0.5,6.3) | 3.0 (-0.6,6.7) |
| **24 months** | | | | |
| 18-24 | 422/505 (84%) | 2,680/3,526 (76%) | 7.6 (-0.7,16.3) | 7.8 (-1.2,17.2) |
| 25-34 | 3,469/3,990 (87%) | 16,745/20,153 (83%) | 3.9 (0.7,7.0) | 4.7 (1.4,8.1) |
| 35-49 | 6,087/6,743 (90%) | 29,147/33,435 (87%) | 3.1 (0.6,5.6) | 3.5 (0.9,6.2) |
| 50+ | 2,225/2,496 (89%) | 13,371/15,674 (85%) | 3.8 (-0.1,7.9) | 4.1 (-0.1,8.5) |
| **36 months** | | | | |
| 18-24 | 234/301 (78%) | 1,445/2,050 (70%) | 7.3 (-3.0,18.2) | 7.0 (-4.2,19.0) |
| 25-34 | 1,982/2,400 (83%) | 9,180/11,550 (79%) | 3.1 (-0.8,7.1) | 3.6 (-0.6,8.0) |
| 35-49 | 3,381/3,892 (87%) | 15,709/18,881 (83%) | 3.7 (0.5,6.9) | 4.2 (0.7,7.8) |
| 50+ | 1,213/1,422 (85%) | 7,234/9,031 (80%) | 5.2 (0.1,10.4) | 5.4 (-0.1,11.1) |

*estimates adjusted for age, sex, urban/rural facility setting, province, WHO stage at ART initiation, years on ART at trial enrolment
