# Supplementary material for: Evaluating the impact of differentiated service delivery (DSD) on retention in care and HIV viral suppression in South Africa: A target trial emulation using routine healthcare data
Source: PLoS Med. 2025 Aug 26;22(8):e1004489. doi: 10.1371/journal.pmed.1004489 (PMC12410879; doi:10.1371/journal.pmed.1004489)
Supplement: S6 Table — (DOCX) [file pmed.1004489.s007.docx]

**Table S6. Age-stratified pooled risk differences for viral suppression**

| **Age group** | **n/N (%) virally suppressed* in DSD** | **n/N (%) virally suppressed in non-DSD** | **Unadjusted Risk Difference comparing DSD vs non-DSD (95% CI)** | **Adjusted** Risk Difference comparing DSD vs non-DSD (95% CI)** |
| --- | --- | --- | --- | --- |
| **12 months** | | | | |
| 18-24 | 531/555 (96%) | 3,257/3,541 (92%) | 3.7 (-4.8,12.6) | 3.3 (-5.9,12.9) |
| 25-34 | 4,171/4,335 (96%) | 19,706/20,765 (95%) | 1.3 (-1.9,4.5) | 1.4 (-2.0,4.8) |
| 35-49 | 7,404/7,627 (97%) | 34,901/36,424 (96%) | 1.3 (-1.2,3.7) | 1.2 (-1.3,3.8) |
| 50+ | 2,717/2,803 (97%) | 16,236/16,913 (96%) | 0.9 (-3.0,4.9) | 1.3 (-2.9,5.6) |
| **24 months** | | | | |
| 18-24 | 329/347 (95%) | 2,013/2,204 (91%) | 3.5 (-7.2,14.8) | 4.1 (-7.6,16.6) |
| 25-34 | 2,780/2,891 (96%) | 12,972/13,762 (94%) | 1.9 (-2.05.9) | 1.3 (-2.8,5.6) |
| 35-49 | 5,002/5,129 (98%) | 23,075/24,119 (96%) | 1.9 (-1.1,4.8) | 1.7 (-1.5,4.9) |
| 50+ | 1,829/1,874 (98%) | 10,575/11,023 (96%) | 1.7 (-3.1,6.6) | 1.6 (-3.5,6.9) |
| **36 months** | | | | |
| 18-24 | 196/200 (98%) | 1,077/1,172 (92%) | 6.1 (-8.1,21.4) | 6.1 (-9.7,23.3) |
| 25-34 | 1,629/1,688 (97%) | 7,161/7,571 (95%) | 1.9 (-3.2,7.2) | 1.6 (-3.8,7.3) |
| 35-49 | 2,811/2,900 (97%) | 12,543/13,078 (96%) | 1.0 (-2.9,5.0) | 0.9 (-3.4,5.2) |
| 50+ | 999/1,019 (98%) | 5,695/5,914 (96%) | 1.7 (-4.7,8.4) | 1.6 (-5.4,8.9) |

*Viral suppression defined as having a viral load of <400 copies/ml

*estimates adjusted for age, sex, urban/rural facility setting, province, WHO stage at ART initiation, years on ART at trial enrolment
