# Supplementary material for: Evaluating the impact of differentiated service delivery (DSD) on retention in care and HIV viral suppression in South Africa: A target trial emulation using routine healthcare data
Source: PLoS Med. 2025 Aug 26;22(8):e1004489. doi: 10.1371/journal.pmed.1004489 (PMC12410879; doi:10.1371/journal.pmed.1004489)
Supplement: S7 Table — (DOCX) [file pmed.1004489.s008.docx]

**Table S7. Sex-stratified pooled risk differences for viral suppression**

| **Age group** | **n/N (%) retained**  **in DSD** | **n/N (%) retained**  **in non-DSD** | **Unadjusted Risk Difference comparing DSD vs non-DSD (95% CI)** | **Adjusted* Risk Difference comparing DSD vs non-DSD (95% CI)** |
| --- | --- | --- | --- | --- |
| **12 months** | | | | |
| Female | 13,090/13,991 (94%) | 65,135/71,970 (91%) | 3.1 (1.3,4.8) | 3.2 (1.3,5.0) |
| Male | 5,493/5,891 (93%) | 28,831/31,816 (91%) | 2.6 (0.0,5.3) | 3.1 (0.3,6.0) |
| **24 months** | | | | |
| Female | 8,678/9,755 (89%) | 43,282/50,782 (85%) | 3.7 (1.7,5.8) | 4.1 (1.9,6.3) |
| Male | 3,525/3,979 (89%) | 18,661/22,006 (85%) | 3.8 (0.6,7.0) | 4.3 (1.0,7.8) |
| **36 months** | | | | |
| Female | 4,924/5,756 (86%) | 23,669/29,119 (81%) | 4.3 (1.7,6.9) | 4.4 (1.5,7.2) |
| Male | 1,886/2,259 (83%) | 9,899/12,393 (80%) | 3.6 (-0.4,7.7) | 4.4 (0.0,8.9) |

*estimates adjusted for age, sex, urban/rural facility setting, province, WHO stage at ART initiation, years on ART at trial enrolment
