# Supplementary material for: Evaluating the impact of differentiated service delivery (DSD) on retention in care and HIV viral suppression in South Africa: A target trial emulation using routine healthcare data
Source: PLoS Med. 2025 Aug 26;22(8):e1004489. doi: 10.1371/journal.pmed.1004489 (PMC12410879; doi:10.1371/journal.pmed.1004489)
Supplement: S8 Table — (DOCX) [file pmed.1004489.s009.docx]

**Table S8. Sex-stratified pooled risk differences for viral suppression**

| **Age group** | **n/N (%) virally suppressed in DSD** | **n/N (%) virally suppressed in non-DSD** | **Unadjusted Risk Difference comparing DSD vs non-DSD (95% CI)** | **Adjusted* Risk Difference comparing DSD vs non-DSD (95% CI)** |
| --- | --- | --- | --- | --- |
| **12 months** | | | | |
| Female | 10,532/10,861 (97%) | 51,790/54,002 (96%) | 1.1 (-1.0,3.1) | 1.1 (-1.0,3.3) |
| Male | 4,291/4,459 (96%) | 22,310/23,641 (94%) | 1.9 (-1.2,5.0) | 1.8 (-1.5,5.2) |
| **24 months** | | | | |
| Female | 7,106/7,307 (97%) | 34,313/35,880 (96%) | 1.6 (-0.8,4.1) | 1.5 (-1.2,4.1) |
| Male | 2,834/2,934 (97%) | 14,322/15,228 (94%) | 2.5 (-1.3,6.5) | 2.2 (-1.9,6.3) |
| **36 months** | | | | |
| Female | 4,119/4,237 (97%) | 18,889/19,673 (96%) | 1.2 (-2.0,4.5) | 1.2 (-2.3,4.8) |
| Male | 1,516/1,570 (97%) | 7,587/8,062 (94%) | 2.5 (-2.8,7.8) | 1.9 (-3.8,7.7) |

*estimates adjusted for age, sex, urban/rural facility setting, province, WHO stage at ART initiation, years on ART at trial enrolment
