# Supplementary material for: Evaluating the impact of differentiated service delivery (DSD) on retention in care and HIV viral suppression in South Africa: A target trial emulation using routine healthcare data
Source: PLoS Med. 2025 Aug 26;22(8):e1004489. doi: 10.1371/journal.pmed.1004489 (PMC12410879; doi:10.1371/journal.pmed.1004489)
Supplement: S9 Table — Net outcomes of those not retained within the trial outcome window period. (DOCX) [file pmed.1004489.s010.docx]

**Table S9: Patient outcomes including reported TIER.Net outcomes of those not retained within the trial outcome window period**

|  | **All** | **DSD** | **Non-DSD** |
| --- | --- | --- | --- |
| **12 months** |  |  |  |
| Retained | 112,549 (91%) | 18,583 (93.5%) | 93,966 (90.5%) |
| Lost to follow-up | 9,132 (7.4%) | 1,097 (5.5%) | 8,035 (7.7%) |
| Died | 229 (0.2%) | 22 (0.1%) | 207 (0.2%) |
| Transferred out | 1,757 (1.4%) | 179 (0.9%) | 1,578 (1.5%) |
| **24 months** |  |  |  |
| Retained | 74,146 (85.7%) | 12,203 (88.9%) | 61,943 (85.1%) |
| Lost to follow-up | 10,340 (12%) | 1,290 (9.4%) | 9,050 (12.4%) |
| Died | 274 (0.3%) | 38 (0.3%) | 236 (0.3%) |
| Transferred out | 1,761 (2%) | 202 (1.5%) | 1,559 (2.1%) |
| **36 months** |  |  |  |
| Retained | 40,378 (81.5%) | 6,810 (85%) | 33,568 (80.9%) |
| Lost to follow-up | 7,938 (16%) | 1,083 (13.5%) | 6,855 (16.5%) |
| Died | 196 (0.4%) | 22 (0.3%) | 174 (0.4%) |
| Transferred out | 1,015 (2%) | 100 (1.2%) | 915 (2.2%) |
