# Supplementary material for: Evaluating the impact of differentiated service delivery (DSD) on retention in care and HIV viral suppression in South Africa: A target trial emulation using routine healthcare data
Source: PLoS Med. 2025 Aug 26;22(8):e1004489. doi: 10.1371/journal.pmed.1004489 (PMC12410879; doi:10.1371/journal.pmed.1004489)
Supplement: S10 Table — (DOCX) [file pmed.1004489.s011.docx]

**S10. RECORD statement checklist**

|  | Item No. | STROBE items | Location in manuscript where items are reported | RECORD items | Location in manuscript where items are reported |
| --- | --- | --- | --- | --- | --- |
| Title and abstract |  |  |  |  |  |
|  | 1 | (a) Indicate the study’s design with a commonly used term in the title or the abstract (b) Provide in the abstract an informative and balanced summary of what was done and what was found | (a) title  (b) abstract | RECORD 1.1: The type of data used should be specified in the title or abstract. When possible, the name of the databases used should be included. RECORD 1.2: If applicable, the geographic region and timeframe within which the study took place should be reported in the title or abstract. RECORD 1.3: If linkage between databases was conducted for the study, this should be clearly stated in the title or abstract. | 1.1 title specifies routine data; abstract specifies database  1.2 Country specified in title; restriction to three SA provinces reported in abstract  1.3 Not applicable |
| Introduction |  |  |  |  |  |
| Background rationale | 2 | Explain the scientific background and rationale for the investigation being reported | Introduction paragraphs 1-3 |  |  |
| Objectives | 3 | State specific objectives, including any prespecified hypotheses | Introduction paragraph 4 |  |  |
| Methods |  |  |  |  |  |
| Study Design | 4 | Present key elements of study design early in the paper | Methods paragraph 1 |  |  |
| Setting | 5 | Describe the setting, locations, and relevant dates, including periods of recruitment, exposure, follow-up, and data collection | Study site setting and location: Methods- Study sites and population, paragraph 1 and Table S1;  Trial time periods: Methods-Development of multiple trial emulations, paragraph 1 |  |  |
| Participants | 6 | (a) Cohort study - Give the eligibility criteria, and the sources and methods of selection of participants. Describe methods of follow -up Case -control study - Give the eligibility criteria, and the sources and methods of case ascertainment and control selection. Give the rationale for the choice of cases and controls Cross -sectional study - Give the eligibility criteria, and the sources and methods of selection of participants (b) Cohort study - For matched studies, give matching criteria and number of exposed and unexposed Case -control study - For matched studies, give matching criteria and the number of controls per case | (a) Eligibility criteria: Methods- Study sites and population, paragraph 1; Methods- Target trial emulation, paragraph 2; Table 2; | RECORD 6.1: The methods of study population selection (such as codes or algorithms used to identify subjects) should be listed in detail. If this is not possible, an explanation should be provided. RECORD 6.2: Any validation studies of the codes or algorithms used to select the population should be referenced. If validation was conducted for this study and not published elsewhere, detailed methods and results should be provided. RECORD 6.3: If the study involved linkage of databases, consider use of a flow diagram or other graphical display to demonstrate the data linkage process, including the number of individuals with linked data at each stage. | 6.1 Eligibility criteria is listed in Methods- Study sites and population, paragraph 1; Methods- Target trial emulation, paragraph 2; Table 2. A flow diagram presenting the cohort is presented in Figure S1  6.2 No validation studies were conducted.  6.2 Not applicable |
| Variables | 7 | Clearly define all outcomes, exposures, predictors, potential confounders, and effect modifiers. Give diagnostic criteria, if applicable . | Outcomes defined in Methods-Outcomes, paragraphs 1-3  DSD models explained in Table 1  Potential confounders described Methods-Statistical analysis, paragraph 1 | RECORD 7.1: A complete list of codes and algorithms used to classify exposures, outcomes, confounders, and effect modifiers should be provided. If these cannot be reported, an explanation should be provided. | The complete list of codes and algorithms is not included but explanation of the methods to define each of these is presented in the text. |
| Data sources/ measurement | 8 | For each variable of interest, give sources of data and details of methods of assessment (measurement). Describe comparability of assessment methods if there is more than one group | All data derive from TIER.Net as indicated in line |  |  |
| Bias | 9 | Describe any efforts to address potential sources of bias | Methods- Target trial emulation, paragraph 1; Methods-Development of multiple trial emulations  , paragraph 1; Methods-Statistical analysis |  |  |
| Study size | 10 | Explain how the study size was arrived at | Methods- Study sites and population, paragraph 1 |  |  |
| Quantitative variables | 11 | Explain how quantitative variables were handled in the analyses. If applicable, describe which groupings were chosen, and why | Table 2, discussion on CD4 vs. WHO stage in Methods-Statistical analysis |  |  |
| Statistical methods | 12 | (a) Describe all statistical methods, including those used to control for confounding (b) Describe any methods used to examine subgroups and interactions (c) Explain how missing data were addressed (d) Cohort study - If applicable, explain how loss to follow - up was addressed Case -control study - If applicable, explain how matching of cases and controls was addressed Cross -sectional study - If applicable, describe analytical methods taking account of sampling strategy (e) Describe any sensitivity analyses | (a-e) Methods-Statistical analysis |  |  |
| Data access and cleaning methods |  |  |  | RECORD 12.1: Authors should describe the extent to which the investigators had access to the database population used to create the study population. RECORD 12.2: Authors should provide information on the data cleaning methods used in the study. | Data access covered under the Methods-Ethics approval section |
| Linkage |  |  |  | RECORD 12.3: State whether the study included person -level, institutional -level, or other data linkage across two or more databases. The methods of linkage and methods of linkage quality evaluation should be provided. | Not applicable. |
| Results |  |  |  |  |  |
| Participants | 13 | (a) Report the numbers of individuals at each stage of the study (e.g., numbers potentially eligible, examined for eligibility, confirmed eligible, included in the study, completing follow -up, and analysed) (b) Give reasons for non - participation at each stage. (c) Consider use of a flow diagram | Reported in Figures 2 and S1 | RECORD 13.1: Describe in detail the selection of the persons included in the study (i.e., study population selection) including filtering based on data quality, data availability and linkage. The selection of included persons can be described in the text and/or by means of the study flow diagram. | Reported in Figures 2 and S1 |
| Descriptive data | 14 | (a) Give characteristics of study participants (e.g., demographic, clinical, social) and information on exposures and potential confounders (b) Indicate the number of participants with missing data for each variable of interest (c) Cohort study - summarise follow -up time (e.g., average and total amount) | (a) Tables 3 and S2  (b) Tables 3 and S2  (c) Total client trials with outcomes and insufficient follow up time presented in Figure 3 |  |  |
| Outcome data | 15 | Cohort study - Report numbers of outcome events or summary measures over time Case -control study - Report numbers in each exposure category, or summary measures of exposure Cross -sectional study - Report numbers of outcome events or summary measures | Figures 3, 4 and 5 |  |  |
| Main results | 16 | (a) Give unadjusted estimates and, if applicable, confounder - adjusted estimates and their precision (e.g., 95% confidence interval). Make clear which confounders were adjusted for and why they were included (b) Report category boundaries when continuous variables were categorized (c) If relevant, consider translating estimates of relative risk into absolute risk for a meaningful time period | Table S3 |  |  |
| Other analyses | 17 | Report other analyses done — e.g., analyses of subgroups and interactions, and sensitivity analyses | Methods-Statistical analysis and  Tables S5-S8 |  |  |
| Discussion |  |  |  |  |  |
| Key results | 18 | Summarise key results with reference to study objectives | Discussion, paragraph 1 |  |  |
| Limitations | 19 | Discuss limitations of the study, taking into account sources of potential bias or imprecision. Discuss both direction and magnitude of any potential bias | Discussion, paragraphs 4 and 5. | RECORD 19.1: Discuss the implications of using data that were not created or collected to answer the specific research question(s). Include discussion of misclassification bias, unmeasured confounding, missing data, and changing eligibility over time, as they pertain to the study being reported. | Discussion, paragraphs 4 and 5. |
| Interpretation | 20 | Give a cautious overall interpretation of results considering objectives, limitations, multiplicity of analyses, results from similar studies, and other relevant evidence | Discussion, paragraph 6 |  |  |
| Generalisability | 21 | Discuss the generalisability (external validity) of the study results | Supplementary information |  |  |
| Other Information |  |  |  |  |  |
| Funding | 22 | Give the source of funding and the role of the funders for the present study and, if applicable, for the original study on which the present article is based | Supplementary information |  |  |
| Accessibility of protocol, raw data, and programming code |  |  |  | RECORD 22.1: Authors should provide information on how to access any supplemental information such as the study protocol, raw data, or programming code | Supplementary information. |

*Reference: Benchimol EI, Smeeth L, Guttmann A, Harron K, Moher D, Petersen I, Sørensen HT, von Elm E, Langan SM, the RECORD Working Committee. The REporting of studies Conducted using Observational Routinely-collected health Data (RECORD) Statement. PLoS Medicine 2015; in press. *Checklist is protected under Creative Commons Attribution (CC BY) license.
